# Epidemiology of Bladder Cancer

**DOI:** 10.3390/medsci8010015

**Published:** 2020-03-13

**Authors:** Kalyan Saginala, Adam Barsouk, John Sukumar Aluru, Prashanth Rawla, Sandeep Anand Padala, Alexander Barsouk

**Affiliations:** 1 Plains Regional Medical Group Internal Medicine, Clovis, NM 88101, USA; 2 Hillman Cancer Center, University of Pittsburgh, Pittsburgh, PA 15232, USA; 3 Senior Research Associate, Beth Israel Deaconess Medical Center, Harvard Medical School, Boston, MA 02212, USA; 4 Department of Medicine, Sovah Health, Martinsville, VA 24112, USA; 5 Department of Medicine, Nephrology, Augusta University, Medical College of Georgia, Augusta, GA 30912, USA; 6 Hematologist-Oncologist, Allegheny Health Network, Pittsburgh, PA 15212, USA

**Keywords:** bladder cancer, epidemiology, incidence, prevalence, mortality, prevention, risk factors

## Abstract

Based on the latest GLOBOCAN data, bladder cancer accounts for 3% of global cancer diagnoses and is especially prevalent in the developed world. In the United States, bladder cancer is the sixth most incident neoplasm. A total of 90% of bladder cancer diagnoses are made in those 55 years of age and older, and the disease is four times more common in men than women. While the average 5-year survival in the US is 77%, the 5-year survival for those with metastatic disease is a measly 5%. The strongest risk factor for bladder cancer is tobacco smoking, which accounts for 50–65% of all cases. Occupational or environmental toxins likewise greatly contribute to disease burden (accounting for an estimated 20% of all cases), though the precise proportion can be obscured by the fact bladder cancer develops decades after exposure, even if the exposure only lasted several years. Schistosomiasis infection is the common cause of bladder cancer in regions of Africa and the Middle East and is considered the second most onerous tropical pathogen after malaria. With 81% of cases attributable to known risk factors (and only 7% to heritable mutations), bladder cancer is a prime candidate for prevention strategies. Smoking cessation, workplace safety practices, weight loss, exercise and schistosomiasis prevention (via water disinfection and mass drug administration) have all been shown to significantly decrease the risk of bladder cancer, which poses a growing burden around the world.

## 1. Introduction

Cancer of the bladder, also known as urological cancer or urinary bladder cancer, is the 10th most common cancer in the world, and its incidence is steadily rising worldwide, especially in developed nations [1,2]. The bladder is a hollow organ in the lower abdomen whose main purpose is to store urine received from the kidneys (via the ureter) until micturition. Specialized transitional epithelial cells lining the urinary bladder and urinary tract, known as urothelial cells, accommodate the volume of urine produced by flattening under pressure. The bladder is also lined with smooth muscle that can relax to accommodate greater volumes, as well as contract (under voluntary or reflex control) to expel urine down the urethra and out of the body [3]. The urothelial cells lining the bladder and urinary tract are constantly exposed to environmental, potentially mutagenic agents that are filtered into the urine by the kidneys [4]. Unsurprisingly, 90% of bladder cancer cases, especially those in the developed world, arise from these urothelial cells, mostly in the bladder but on rare occasions in the urinary tract as well. While localized forms of urothelial cancer carry an excellent prognosis, if the smooth muscle is invaded, survival rates drop significantly. Squamous cell bladder cancer, which accounts for the remaining 10% of cases, is more prevalent in Africa and likely associated with the protozoan infection schistosomiasis [5].

Most bladder cancers can be traced back to exposure to environmental and occupational chemicals, the largest of which by far is tobacco smoke. Greater tobacco smoke and occupational exposure in men may help explain the 4-fold gender discrepancy in bladder cancer incidence [1]. The relative risk of bladder cancer following tobacco consumption is second only to that of lung cancer [6]. Although tobacco consumption has fallen over past decades in the US [7], bladder cancer mortality has remained consistent, likely due to a “lag effect” from previous tobacco consumption [8]. In fact, approximately 80% of cases of bladder cancer are diagnosed in adults age 65 or older, reflecting a disease course that requires decades of exposure or develops decades after exposure [4]. Heritable genetic predispositions have also been implicated in approximately 7% of bladder cancer cases [9].

Bladder cancer is usually first suspected due to hematuria and then identified with a cystoscopy, a telescopic endoscopy of the bladder, transabdominal ultrasound, and/or computer tomography (CT) urography. As many as 7 out of every 10 cases of bladder cancer are detected in early stages, thus allowing for resection and improved survival. Non-muscle-invasive bladder cancers (NMIBC) are typically removed by transurethral resection. Alternatively, a cystoscopy plus biopsy procedure may be used for certain resections. Intravesicular cytotoxic therapy may be added for high-risk cases. Meanwhile, for the 30% of patients who present with muscle-invasive bladder cancer (MIBC), neoadjuvant or adjuvant chemotherapy is considered the standard to lower the risk of recurrence, and radical cystectomy is the mainstay surgical treatment. External beam radiation may also be used. For the treatment of metastatic disease (which currently only has a 5% 5-year survival rate), platinum chemotherapy remains the standard, though novel immunotherapies, namely checkpoint inhibitors, are growing in popularity as treatment options in first-line and beyond [4]. 

While survival rates have improved with earlier diagnosis, robotic surgical techniques, and the introduction of immunotherapy, bladder cancer remains a significant and rising contributor to cancer burden worldwide, especially in developed nations [1]. A better understanding of the epidemiology and risk factors underlying bladder cancer is crucial for its prevention.

## 2. Epidemiology

### 2.1. Incidence

**Figure 1:**
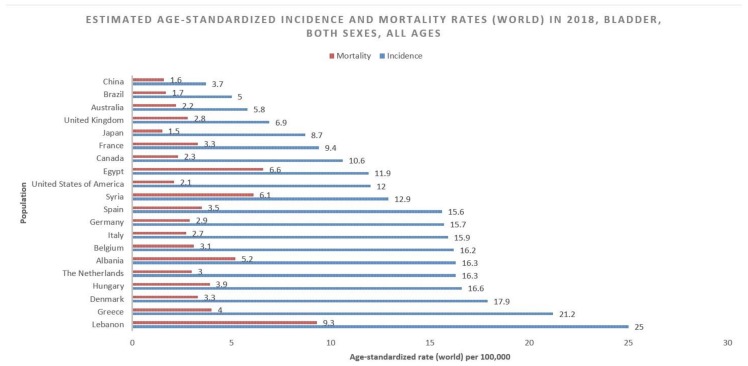
Bar chart showing estimated age-standardized incidence and mortality rates (world) in 2018, bladder cancer, all sexes, all ages. Data obtained from Globocan 2018 [2].

**Figure 2:**
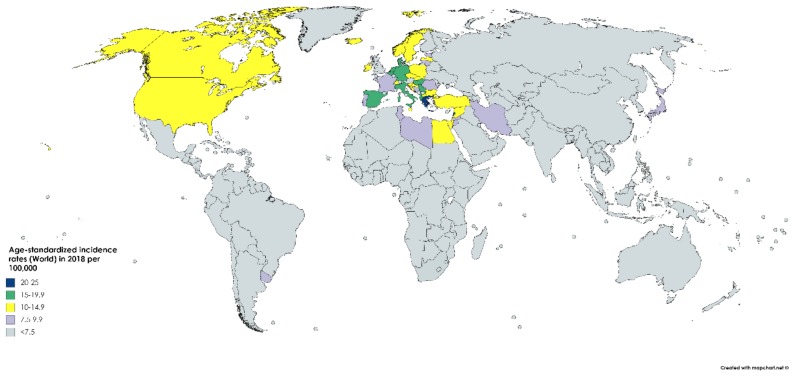
Map showing estimated age-standardized incidence rates (ASR) for bladder cancer worldwide in 2018, all sexes, including all ages. Created with mapchart.net. Data obtained from Globocan 2018 [2].

According to GLOBOCAN data, an estimated 550,000 people were diagnosed with bladder cancer in 2018. This accounts for roughly 3% of all new cancer diagnoses. Nations with the highest rates of bladder cancer are largely found in Southern and Western Europe as well as North America. While Greece has the highest rate of bladder cancer among men, Lebanon has the highest rate among women. Nevertheless, the region with the highest rate of bladder cancers among women is Southern Europe (same as among men), where an estimated 26.5/100,000 men and 5.5/100,000 women develop the disease every year (Figure 1). The regions with the lowest incidence of bladder cancer include Middle Africa, Central America, and West Africa, largely composed of nations that are below average on the human development index (HDI), possibly due to lower industrial chemical exposure and limited access to tobacco [1]. In fact, bladder cancer incidence has been found to be positively correlated with HDI and, to a lesser extent, GDP (Gross Domestic Product) per capita (Figure 2) [6]. 

An estimated 80,500 cases of bladder cancer were diagnosed in the United States (US) in 2019, representing 4.6% of all cancer diagnoses (greater than the global average). This makes bladder cancer the sixth most common cancer diagnosis in the US. The incidence of bladder cancer in the US rose from 19.3/100,000 in 1975 to a peak of 21.6/100,000 in 1987 and has since steadily declined over the past decades to 18.1/100,000 in 2016 [10]. In many European nations, such as Germany and Bulgaria, incidence rates of bladder cancer have continued to rise, and are expected to rise even further, due to a greater prevalence of smoking and an aging population. Other nations have made significant progress in the prevention, with a nearly 11% reduction in incidence in New Zealand over the past 10 years [11].

Bladder cancer is over four times more common in men than women, with a respective incidence of 9.6/100,000 among men and 2.4/100,000 among women worldwide. Among men, bladder cancer is the sixth most incident and ninth most deadly neoplasm [1]. This gender discrepancy is likely most attributable to gender differences in smoking tobacco, which may also help explain why cancer is rising among women in the developed world. In the United States, as many as 50%-65% of cases of bladder cancer are estimated to be attributable to tobacco smoke, the largest risk factor for the disease [11]. The attributable risk to smoking has recently grown to be similar among men and women [8]. 

Ninety% of new bladder cancer diagnoses in the US are in people 55 years of age or older, and the average age of diagnosis is 73 [12].

### 2.2. Mortality

**Figure 3:**
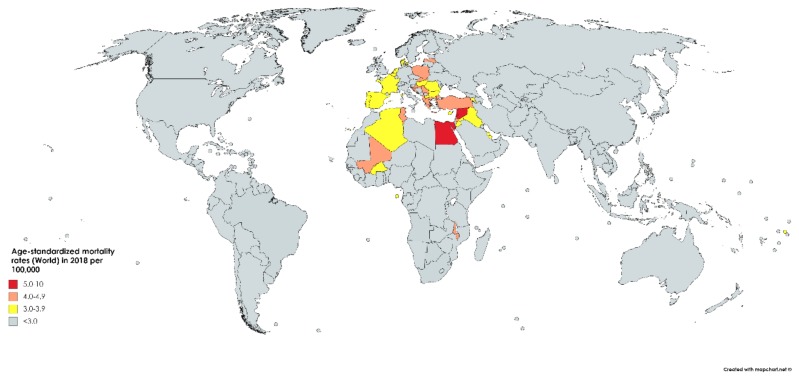
Map showing estimated age-standardized mortality rates (ASR) for bladder cancer worldwide in 2018, all sexes, including all ages. Created with mapchart.net. Data obtained from Globocan 2018 [2].

While bladder cancer is the 10th most common neoplasm throughout the world, it is the 13th most deadly, estimated to have claimed nearly 200,000 lives in 2018. This constitutes 2.1% of all cancer deaths. Mortality rates reflect incidence rates in terms of gender disparity, with a mortality of 3.2/100,000 men, which is roughly four times greater than that of women worldwide (0.9/100,000). The cumulative risk of dying from bladder cancer between birth and the age of 74 is 0.29% among men and 0.08% among women. Mortality is greatest in North and East Africa and the Middle-East, where schistosomiasis infection leads to high incidence rates. The highest mortality is in Egypt at 6.6/100,000 (Figure 3) [1].

An estimated 17,600 people expired from bladder cancer in the US in 2019. This figure represents 2.9% of all cancer deaths, which is lower than the incidence as a proportion of all cancers (4.6%), reflecting above-average survival for the disease (relative to other neoplasms). Although incidence has fallen over the past decades, bladder cancer mortality has remained steadfast at 4.4/100,000 since 1987. Nevertheless, this represents an improvement from the earliest recorded mortality in the US of 5.5/100,000 in 1975. The drop in mortality has stagnated over the past decades in spite of improved early diagnostic, resection and targeted treatment techniques [10]. Bladder cancer is the eighth most common cause of cancer death in the US [12].

Around the world, there has been a substantial decrease in bladder cancer mortality, despite the increase in incidence. However, mortality rates have increased in several countries, namely Iceland, Ecuador, and the Philippines [6]. 

### 2.3. Survival

**Figure 4:**
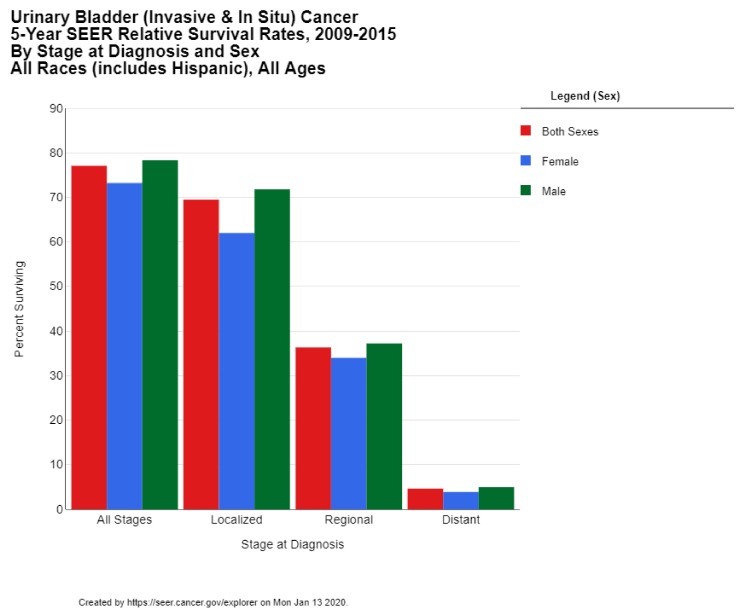
Urinary Bladder (Invasive & In Situ) Cancer 5-Year SEER Relative Survival Rates, 2009–2015 By Stage at Diagnosis and Sex. Data source: US Mortality Files, National Center for Health Statistics, CDC [13].

The 5-year survival rate for bladder cancer in the US is 77.1%. While the 5-year survival is 95.8% among those cases diagnosed in situ (which comprise 51% of all diagnoses), the survival rate drops down to 69.5% for localized disease (34% of all cases), 36.3% of regional disease (7% of all cases), and only 4.6% survival for metastatic disease (5% of all cases) (Figure 4) [13]. These statistics reflect the successes of early diagnosis, as well as the poor prognosis for metastatic bladder cancer. The 5-year survival rate in the US has risen over the past 4 decades from 71.9% for diagnoses in 1975 to 79.3% for diagnoses in 2011 [10]. The 10-year survival rate in the US is 70%, and the 15-year survival is 65% [12]. 

## 3. Etiology

Urothelial cell bladder cancer accounts for 90% of bladder cancer cases worldwide and is especially common in developed nations. This subtype is highly associated with chemical exposure, such as occupational exposure or tobacco smoke, due to urothelial direct exposure. These cancers migrate beyond the urothelium and invade the submucosa, lamina propria, muscle and serous layers of the bladder. They may also spread directly to adjacent pelvic structures, including the prostate, urethra, uterus, and vagina. Lymphatic metastasis occurs via the obturator, presacral, iliac and para-aortic lymph nodes, while hematogenous spread usually results in metastases to the liver, lungs, bones, and adrenal glands and is associated with a poor prognosis [4]. 

Five percent of worldwide bladder cancer cases arise from squamous cells, and these cases are more incident in Africa, likely due to schistosomiasis, a protozoal infection which promotes inflammation [14]. The remaining 5% are composed of rare subtypes such as adenocarcinoma, sarcoma, and metastases to the bladder [4].

## 4. Risk Factors

### 4.1. Gender

Across the world, bladder cancer is around four times more likely to be diagnosed in men than women. Mortality is likewise around four times greater in men [1]. While much of this discrepancy can be attributed to differential rates of tobacco smoking, the relative risk of bladder cancer death among smokers is still higher among men than women (3.0 vs. 2.4) [15]. Certain countries where tobacco smoking is culturally prevalent among women have particularly high rates of bladder cancer, with Lebanon having the highest incidence among women worldwide [1]. Other hypothesized factors that predispose men include occupational chemical exposure, and alcohol and red-meat consumption. In both males and females, the onset of bladder cancer is about 6 years earlier for current smokers than for current non-smokers [16]. 

### 4.2. Age

Bladder cancer is predominantly a disease of older adults, with 90% of diagnoses made in those over 55, and 80% of diagnoses in those over 65 in the US. The average age for bladder cancer diagnosis in the US is 73 [12]. This is older than the average age of cancer diagnosis (65–70 years of age), indicating a disease course that requires decades post-exposure to mutagens to overcome cellular tumor-suppressor mechanisms and culminate in carcinogenesis. Although extremely rare, bladder cancer can be seen in children and young adults, where it usually presents with low-grade, noninvasive disease [17].

### 4.3. Hereditary and Genetic Factors

While studies have failed to uncover major germline genetic players underlying sporadic bladder cancer, many genetic loci have been found via genome wide association studies to have a modest association with an increased susceptibility to bladder cancer [18]. Among these are MYC, a cell-signaling molecule and common oncogene [19,20], NAT2, a slow acetylator which functions to detoxify aromatic amines [21], and GSTM1, an enzyme involved in the detoxification of other environmental carcinogens [22,23]. The latter two (NAT2 and GSTM1) also seem to have a synergistic carcinogenic interaction with tobacco smoking [24]. 

While bladder cancer is typically not considered as hereditary, certain cancer symptoms predispose one’s risk to bladder cancer. One example is Cowden’s syndrome, a hereditary defect in tumor-suppressor gene PTEN which predisposes to a wide variety of neoplasms, including transitional and squamous cell urothelial cancer [25]. Another is Lynch syndrome, a defect in DNA mismatch repair typically associated with non-polyposis colorectal cancer, but which also increases the risk of bladder cancer [26]. These patients are particularly good candidates for checkpoint inhibitor immunotherapy [27]. 

Among somatic mutations, growth factor receptor FGFR has been implicated in up to 20% of recurrent bladder cancer patients, leading to the approval of the tyrosine kinase inhibitor erdafitinib as a later line therapy [28]. As in many neoplasms, p53 mutations are implicated in bladder cancer carcinogenesis and may even have prognostic value [29,30].

### 4.4. Smoking

Smoking tobacco is by far the greatest risk factor for bladder cancer, accounting for approximately 50–65% of new cases each year. Smoking has been shown to increase the risk of bladder cancer by three to four times. The relative risk for bladder cancer mortality due to smoking is second only to lung cancer, which is the number one cause of cancer death in the world [8]. Tobacco smoke contains known carcinogens such as beta-naphthylamine and polycyclic aromatic hydrocarbons. These particles promote inflammation, and their metabolism, in the bladder and throughout the body, culminates in DNA-adduct formation and permanent genetic mutation. Such mutations can activate oncogenes or suppress tumor suppressor genes, promoting carcinogenesis. Certain inherited genotypes associated with abnormal detoxification enzymes have been shown to increase the susceptibility to cancer among those who smoke [31].

The older age of onset of bladder cancer suggests a latency period of approximately 30 years from the initiation of smoking to the cancer diagnosis. However, smoking cessation has been shown to reduce the risk of bladder cancer by approximately 40% within only 1–4 years, and complete return to baseline risk by 20 years, suggesting a non-linear relationship between incidence and pack-years [32]. One study found pure tobacco cigarette smokers (95% confidence interval (CI) 2.9–4.2) were at greater risk than pure pipe smokers (CI 1.2–3.1) or pure tobacco cigar smokers (CI 1.6–3.5). These latter forms of smoking are also associated with a lesser risk of lung and head and neck cancer, likely because they reach lower temperatures than cigarettes, contain fewer chemicals, and result in not as much inhalation of carcinogenic particles [33]. A meta-analysis of 14 studies by Yan et al. showed that there was 22% increased risk of bladder cancer for lifetime secondhand smoking exposure in nonsmoking patients compared with unexposed nonsmoking population [34]. 

### 4.5. Environmental and Occupational Exposure 

The second greatest preventable risk factor for bladder cancer is occupational exposure to carcinogens, including aromatic amines, polycyclic aromatic hydrocarbons, and chlorinated hydrocarbons [35]. These compounds are commonly found in the industrial production of dyes, paint, metal, rubber or petroleum products [32]. Among those in the rubber industry, an increased mortality risk of 253× was reported for those in “storage and shipment” and an increased risk of 159× was reported for those with “general work” in the industry [36]. Other industries implicated in a greater risk of bladder cancer include firefighters, hairdressers, and farmers who use fungicides. Overall, occupational exposures are estimated to be responsible for 18% of bladder cancer cases. While 2 years’ exposure seems to be sufficient to increase one’s risk, the disease often does not develop until decades after exposure, much like with tobacco smoke [37]. 

A large prospective observational study from Chile suggested that exposure to arsenic, a naturally occurring metalloid in air, soil, and water, also increased the risk of bladder cancer [38]. Another study from Finland found that exposure to low concentration Arsenic (0.5 µg/L) and tobacco smoke had a synergistic effect in increasing the risk of bladder cancer [39]. Other carcinogens in the water supply, such as disinfection byproducts or nitrates, along with metals in the diet such as selenium and zinc, could also modify the risk of developing bladder cancer [40].

### 4.6. Alcohol

Several studies have shown that alcohol may slightly increase the risk of developing bladder cancer, though this increase has not been proven to be statistically significant [41]. Alcohol is better implicated in the development of other cancers, such as hepatocellular carcinoma and colorectal carcinoma [42]. 

### 4.7. Red Meat

One recent meta-analysis found that high red meat and processed meat intake increased the risk of bladder cancer by 17% and 10%, respectively [43]. A different review found a 22% increase for processed meats, but no statistically significant increase for red meats [44]. The discrepancy has been attributed to different levels of defining high meat consumption. It has been shown that nitrosamines from nitrates in processed meats directly induce the development of bladder tumors in rodents [45]. Red and processed meat are strongly implicated in colorectal cancer incidence, leading the International Agency for Cancer Research to designate processed meat as a Group 1 carcinogen, and red meat as a probable carcinogen, in 2015 [46]. Meanwhile, the consumption of poultry and pork has not been shown to increase the risk of bladder cancer [47].

### 4.8. Obesity

A meta-analysis of 15 cohort studies found that pre-obesity increases the risk of bladder cancer by 7% while obesity increases that risk by 10%. A linear relationship between BMI (Body Mass Index) and bladder cancer risk was revealed, with a 5 kg/m^2^ increase in weight associated with a 4.2% increased risk of bladder cancer. This risk was found to be independent of smoking, physical activity, alcohol or diet [48]. Although obesity is associated with many forms of cancer, the biological mechanism is not well understood. Obesity increases the production of insulin and insulin-like growth factor-I which modify cell proliferation, angiogenesis, and apoptosis [49]. Obesity also promotes chronic inflammation by altering the levels of cytokines, thereby initiating an immune cascade that ultimately promotes carcinogenesis [50]. Physical activity seems to have a protective effect against bladder cancer independent of obesity [51].

### 4.9. Pathogens 

The protozoan schistosomiasis, which infects roughly 240 million humans worldwide through freshwater exposure, is second only to malaria in the suffering caused by a tropical pathogen, largely due to its associated increased risk of bladder cancer [52]. In fact, squamous cell carcinoma of the bladder is the second most common form of cancer in regions of the Middle-East and Africa where schistosomiasis is endemic (after hepatic cancer, which is also associated with the pathogen) [5]. In these regions, the mean age of incidence of bladder cancer is between 40-49 years of age (as opposed and greater than 70 years of age in most of the developed world). Interestingly, these nations still demonstrate a 4-5-fold greater risk of bladder cancer for men than women, like due to the transmission of schistosomiasis via agricultural activities typically done by men [53]. Schistosomiasis infection of the bladder has been shown to cause infection by bacteria that generate carcinogens such as N-nitroso compounds. The infection also promotes inflammation, inducing endogenous synthesis of N-nitrosamines as well as DNA-damaging free oxygen radicals [14]. This disease course seems to occur far more rapidly than that of tobacco or chemical exposure and can be prevented through parasite control via the anti-helminthic agent, praziquantel [52]. 

## 5. Prevention

A recent meta-analysis found that among bladder cancer cases studied, between 1995 and 2015, 81.8% could be attributed to known preventable causes. Only 7% of bladder cancer cases are predicted to arise from heritable genetic influence [9]. With such a large proportion of cases attributable to known environmental causes, bladder cancer is an optimal candidate for public health prevention interventions.

Tobacco smoking is by far the greatest risk factor for bladder cancer, accounting for 65–50% of all cases in the developed world. Smoking cessation has been shown to reduce the risk of bladder cancer by approximately 40% within only 1–4 years, and complete return to baseline risk by 20 years [8]. If cessation is not possible, cigar or pipe smoking carries less risk of carcinogenesis than cigarette smoking. Second-hand exposure to smoke likewise increases risk and must be avoided [54]. Those with certain genetic mutations are at increased susceptibility to cancer from tobacco smoke [31].

Occupational exposure is the second greatest preventable risk factor for bladder cancer. Precautions should be taken to minimize chemical exposure (via aerosols and contact) among those in the manufacturing, shipping, fire-fighting, and hair-styling industries [32]. Interestingly, farmers, gardeners, teachers and forestry workers, who likely have less regular contact with toxic environmental agents in the workplace and in their surroundings, have been found to be at a significantly lower risk of bladder cancer [55]. 

A diet rich in fruits and vegetables may help prevent bladder cancer, though the extent to which remains controversial. An analysis of several meta-analyses found that higher intake of selenium, Vitamins A, D and E, and folate were all associated with a reduction in bladder cancer incidence [9]. Vitamin E is an anti-inflammatory and antioxidant which may counter damage caused by free radicals. Although found to be protective in the aforementioned meta-analysis, Vitamin E and Selenium were not found to decrease the incidence of bladder cancer in a recent double-blind clinical trial [56]. Vitamin D is endogenously converted into calcitriol, which has been shown to play a role in cancer regulation pathways. Folate is essential to DNA repair and expression and has been shown to prevent cancer formation by maintaining proper nucleotide synthesis and methylation. However, folate has also been implicated in accelerating the progression of already existent tumors by increasing the availability of nucleotides. Likewise, Vitamin A (an antioxidant) has been shown to be protective against bladder cancer in dietary doses but potentially harmful in the much larger doses received from supplements. None of these agents have been proven to decrease bladder cancer risk in clinical trials [9]. 

Physical activity has been shown to have a small protective role against bladder cancer (independent of smoking or BMI), and its effect may be amplified if used as part of a weight-loss program [51]. The inflammation and proliferation signaling caused by obesity has been implicated in a wide variety of cancers. Taking into account its additional deleterious sequelae on the cardiovascular system, obesity is the number one preventable cause of death in the US and the rest of the developed world. 

Disinfecting drinking and bathing water and avoiding freshwater swimming and wading in endemic regions could significantly decrease the risk of schistosomiasis [52]. Mass drug administration of anthelminthic agent praziquantel could disease control and significantly decreases the risk of developing bladder cancer. Vaccines and novel approaches towards life cycle modification are being developed to attempt to control the oft-neglected tropical disease [57].

## 6. Conclusions

Bladder cancer is the 10th most common and 13th most deadly cancer worldwide. Its incidence is steadily rising, especially in Europe and other developed countries, while mortality worldwide is falling thanks to improved prevention, early diagnosis, and treatment. Bladder cancer is approximately four times more common in men than women, and the average age of diagnosis is 73 in the US. Average 5-year survival with bladder cancer is around 77% in the US and is highly dependent on the stage at diagnosis. Metastatic disease, which accounts for only 5% of cases in the US, has a 5-year survival under 5%. The greatest risk factor for the urothelial subtype, which comprises 90% of all cases, is tobacco smoking. Smoking accounts for 50–65% of all bladder cancer cases and increases the risk of the disease by 3-fold. Meanwhile, squamous cell carcinoma of the bladder, which accounts for 5% of cases, is especially common in Africa and the Middle East and largely associated with the protozoal infection schistosomiasis. The second greatest risk factor after smoking is environmental and occupational exposure to carcinogenic chemicals. An estimated 81% of bladder cancer cases can be attributed to preventable risk factors. Prevention strategies, including smoking cessation, responsible workplace safety practices, diet, weight loss, and schistosomiasis prevention, could all significantly lighten the growing worldwide burden of bladder cancer.
